# Association between primary hypothyroidism and metabolic syndrome and the role of C reactive protein: a cross–sectional study from South India

**DOI:** 10.1186/1756-6614-2-2

**Published:** 2009-03-09

**Authors:** Ghanshyam Palamaner Subash Shantha, Anita A Kumar, Vijay Jeyachandran, Deepan Rajamanickam, K Rajkumar, Shihas Salim, Kuyilan Karai Subramanian, Senthilkumar Natesan

**Affiliations:** 1 Department of General Medicine, Sri Ramachandra University, Chennai, India

## Abstract

**Background:**

Hypothyroidism (sub-clinical and overt) and metabolic syndrome are recognized risk factors for atherosclerotic cardiovascular disease. This study is an effort to identify the proposed association between these two disease entities and the risk factors involved in this association.

**Methods:**

A cross – sectional study from a tertiary care teaching hospital in Chennai city, South India. 420 patients with metabolic syndrome (NCEP – ATP III criteria) were included in the study group. 406 appropriately age and sex matched controls having no features of metabolic syndrome (0 out of the 5 criteria) were compared with the study group. The study extended over a 5 year period. TSH, FT4 were measured for both the groups using electrochemiluminescence immuno assay. HsCRP was measured for all the patients in the study group. The baseline characteristics between the groups were compared with Student's't' test. Chi-square test was used to analyze the association between metabolic syndrome and hypothyroidism (overt and sub-clinical). Logistic regression analysis was applied to identify the association between hypothyroidism and the patient characteristics in the study group.

**Results:**

Of the 420 patients in the study group, 240 were females (57.1%), 180 were males (42.9%) with mean age 51 ± 9.4 years. Of the 406 patients in the control group, 216 were females (53.2%), 190 males (46.8%) with mean age 49 ± 11.2 years. In the study group, 92 had sub-clinical hypothyroidism (SCH) (21.9%), 31 were overtly hypothyroid (7.4%) and 297 were euthyroid (70.7%). In the control group 27 patients had sub-clinical hypothyroidism (6.6%), 9 patients had overt hypothyroidism (2.2%) and 370 patients were euthyroid (91.2%). On comparison SCH (P < 0.001) and overt hypothyroidism (P < 0.001) were significantly associated with the study group as compared to the control group. Logistic regression analysis recognized the association between female gender (P = 0.021) and HsCRP (P = 0.014) with sub-clinical hypothyroidism and female gender (P = 0.01) with overt hypothyroidism in the study group.

**Conclusion:**

Hypothyroidism is associated with metabolic syndrome and females are more at risk. Metabolic syndrome patients with a raised HsCRP are at significant risk of having sub-clinical hypothyroidism.

## Background

Metabolic syndrome constitutes a cluster of risk factors characterized by hypertension, atherogenic dyslipidemia, hyperglycemia, prothrombotic and proinflammatory conditions [1]. As early as 1923, Kylin described the clustering of hyperuricemia, hyperinsulinemia and hypertension [2]. In 1988, during the seminal Banting lecture, Reaven described the central role of insulin resistance in syndrome X, which has now become known as the metabolic syndrome [3]. It was also referred to as insulin resistance syndrome by some until 1999, when the WHO named it metabolic syndrome as there was not sufficient evidence to show that all its components were caused by insulin resistance [4].

Metabolic syndrome (MetS) affects approximately one quarter of the population in developed countries. Its presence is a major risk for development of both type 2 diabetes mellitus and atherosclerosis. The prevalence of cardiovascular disease is 2–3 times higher in individuals with metabolic syndrome than in age-matched controls [5]. According to CURES 52 study, hypertension is prevalent in 20% of Chennai urban population [6]. Among these hypertensive patients, the prevalence of other components of metabolic syndrome was: diabetes in 31.8%, impaired glucose tolerance in 17.9%, hypercholesterolemia in 38.8%, hypertryglyceridemia in 38%, abdominal obesity in 64.3% and general obesity in 40% [6]. The Jaipur Heart Watch Studies have reported that in urban Indian populations, age-adjusted prevalence of metabolic syndrome was 18.4% in men, 30.9% in women, and 24.9% overall [7].

Sub-clinical hypothyroidism (SCH) and overt hypothyroidism are recognized risk factors for atherosclerotic cardiovascular disease, hyperlipidemia, low grade inflammation and hypercoagulability [8-10]. There is scanty data on the prevalence and the various associations of SCH and overt hypothyroidism in the South Indian general population.

As metabolic syndrome and hypothyroidism are independent risk factors for the same disease process, namely cardiovascular disease, it is possible that patients suffering from both these disease entities may have a compounded risk. Our study is an effort to investigate the proposed association between these two disease entities and to identify the factors that increase the risk of this association.

Systemic inflammation measured by high sensitivity C reactive protein (HsCRP) is a known risk factor for cardiovascular disease. Association between metabolic syndrome and HsCRP has been clearly identified and a recent Japanese study has redefined metabolic syndrome with HsCRP as a component in this definition [11,12]. In this study, we have tried to answer the question whether metabolic syndrome patients with a raised HsCRP have an increased risk of having hypothyroidism.

## Methods

This cross sectional study was conducted in an outpatient general medicine clinic of a tertiary care teaching hospital in Chennai, South India. 420 patients with metabolic syndrome (MetS) who fulfilled the NCEP-ATP III criteria (3 out of 5 criteria positive namely blood pressure > or = 130/85 mm hg or on antihypertensive medications, fasting plasma glucose > 110 mg/dl or on anti-diabetic medications, fasting triglycerides > 150 mg/dl, HDL < 40 mg/dl in males and <50 mg/dl in females, waist circumference > 102 cms in men and 88 cms in women) were included in the study group [13]. 406 patients who had no features of metabolic syndrome (0 out of 5 criteria for metabolic syndrome) were included in the control group. The study extended over a 5 year period (June 2003 to June 2008). Patients with liver disorders, renal disorders, congestive cardiac failure, pregnant women, patients on oral contraceptive pills, statins and other medications that alter thyroid functions and lipid levels were excluded from the study. Patients who were diagnosed as having hyperthyroidism, sub-clinical hyperthyroidism and those who are under treatment for any thyroid related disorder were excluded from the study. From both the study and the control group baseline demographic data were collected and a detailed physical examination was performed. Blood pressure was measured over the right arm with the patient lying supine. 3 readings were taken and a mean value of the 3 readings was taken as the final recording. Waist circumference was measured at the plane between anterior superior iliac spines and lower costal margin at the narrowest part of the waistline while the patient was standing and during expiration. Fasting blood samples were obtained (venous blood samples taken after overnight fast of a minimum of 8 hrs); glucose, total cholesterol, HDL cholesterol and triglyceride levels were determined. LDL was calculated using Friedwald formula [14]. Serum TSH and FT4 measurements were made using Roche elecsys modular analytics E 170 using electrochemiluminescence immuno assay (ECLIA method). The analytical sensitivity of TSH was 0.005 μIU/ml and for FT4 was 0.023 ng/dl. Normal range for TSH was (0.27–4.2) μIU/ml and for FT4 was (0.93–1.7) ng/dl. A high serum TSH level (range between 4.2 μIU/ml to 10 μIU/ml) and a normal free thyroxine (FT4) level were required for the diagnosis of sub-clinical hypothyroidism (SCH) [15]. Patients with high TSH (> 10 μIU/ml) and low FT4 levels (< 0.93 ng/dl) were classified as being overt hypothyroid. Patients with normal TSH and FT4 were considered euthyroid. Plasma levels of HsCRP were assessed using validated high sensitivity assay (Dade Behring N high sensitivity CRP assay, Marburg, Germany), with a coefficient of variation of 3.6%. Reference range < 3.0 mg/l. Informed consent was obtained from all the study participants and the ethics committee of our tertiary care hospital approved the study. Baseline characteristics of the study participants were expressed in mean ± SD and percentage. Student's 't' test was used to analyze differences in baseline characteristics between the study group and the control group. Chi-square test was used to analyze the association between metabolic syndrome and hypothyroidism (overt and sub-clinical). Associations between patient characteristics (age, gender, smoking status, alcohol consumption, mean systolic blood pressure, mean diastolic blood pressure, waist circumference, total cholesterol, HDL cholesterol, LDL cholesterol, triglycerides, fasting blood sugar, HsCRP) and hypothyroidism (overt and sub-clinical) in the study group were analyzed using multiple logistic regression. P-value of < 0.05 was considered statistically significant. Statistical analysis was performed using SPSS windows version 16.0 software (SPSS Inc., Chicago, Illinois).

## Results

Of the 420 patients in the study group, 240 were females (57.1%) and 180 were males (42.9%) with mean age 51 ± 9.4 years. The control group (n = 406) had 216 females (53.2%) and 190 males (46.8%) with mean age 49 ± 11.2 years. The baseline characteristics of the two groups are depicted in (Table 1). The two groups were similar with respect to age, sex, smoking and alcohol use (P > 0.05). However mean systolic pressure, diastolic pressure, waist circumference, fasting blood sugar, total cholesterol, LDL cholesterol, triglycerides, TSH and HsCRP values were significantly higher in the study group compared to the control group. HDL cholesterol and FT4 values were significantly lower in the study group (Table 1).

**Table 1:** Patient Characteristics

	**MetS Group (n = 420)**	**Control group (n = 406)**	**Significance (P)**
Age (year)	51 ± 9.4 years	49 ± 11.2 years	0.071

Gender (n)			
Males	180	190	0.51
Females	240	216	

Smoking (n)	169	190	0.321

Alcohol (n)	148	153	0.256

Systolic BP (mmhg)	140.8 ± 5.4	121.3 ± 3.1	0.001

Diastolic BP (mmhg)	90.7 ± 8.5	84 ± 5.73	0.001

Waist circumference (cms)	100.1 ± 5.72	84.2 ± 6.91	0.001

Total cholesterol (mg/dl)	225.9 ± 26.7	191.4 ± 31.2	0.012

HDL cholesterol (mg/dl)	40.2 ± 8.9	46.5 ± 5.76	0.031

LDL cholesterol (mg/dl)	135.9 ± 21.5	109.3 ± 18.6	0.028

Triglycerides (mg/dl)	174.7 ± 58.9	121.6 ± 67.2	0.0001

Fasting Blood Sugar (mg/dl)	109.8 ± 12.8	98.5 ± 9.87	0.031

FT4 (ng/dl)	1.12 ± 0.7	1.3 ± 0.6	0.041

TSH (μIU/ml)	6.1 ± 4.21	5.3 ± 4.89	0.023

HsCRP (mg/l)	2.8 ± 1.9	0.9 ± 1.23	0.001

In the study group, 92 had sub-clinical hypothyroidism (21.9%), 31 were overtly hypothyroid (7.4%) and 297 were euthyroid (70.7%). In the control group 27 had sub-clinical hypothyroidism (6.6%), 9 had overt hypothyroidism (2.2%) and 370 were euthyroid (91.2%). Hence on comparison SCH (P < 0.001) and overt hypothyroidism (P < 0.001) were significantly associated with the study group as compared to the control group.

Logistic regression analysis recognized the association between female gender (P = 0.021, CI: 1.912–12.112) and HsCRP (P = 0.014, CI: 1.587–6.482) with sub-clinical hypothyroidism (Table 2); and female gender (P = 0.01, CI: 1.912 – 2.241) was associated with hypothyroidism in the study group (Table 3).

**Table 2:** Association between patient characteristics and sub-clinical hypothyroidism in the study group (Logistic regression analysis)

	**Odds ratio**	**Confidence interval**	**Significance**
Gender	6.201	(1.912–12.112)	0.021

Smoking status	0.221	(0.276–4.511)	0.312

Age	0.421	(0.451–1.112)	0.133

Alcohol consumption	0.312	(0.812–3.116)	0.124

Waist circumference	2.112	(0.621–1.863)	0.41

Systolic blood pressure	0.781	(0.539–1.221)	0.061

Diastolic blood pressure	0.314	(0.351–3.951)	0.276

Total cholesterol	1.817	(1.541–1.921)	0.213

Triglycerides	0.154	(0.143–1.112)	0.062

HDL cholesterol	1.217	(0.427–4.332)	0.344

Fasting Blood Sugar	0.312	(0.811–2.435)	0.371

HsCRP	1.84	(1.587–6.482)	0.014

**Table 3:** Association between patient characteristics and overt hypothyroidism in the study group (Logistic regression analysis)

	**Odds ratio**	**Confidence interval**	**Significance**
Gender	2.121	(1.912 – 2.241)	0.01

Smoking status	0.319	(0.112–2.311)	0.256

Age	0.223	(0.112–1.106)	0.141

Alcohol consumption	0.418	(0.817–3.222)	0.067

Waist circumference	2.112	(0.221–2.167)	0.296

Systolic blood pressure	0.265	(0.522–2.977)	0.067

Diastolic blood pressure	1.911	(0.481–3.219)	0.413

Total cholesterol	0.144	(0.544–3.227)	0.129

Triglycerides	0.671	(0.511–1.371)	0.133

HDL cholesterol	1.117	(0.283–2.947)	0.216

Fasting blood Sugar	0.591	(0.687–1.395)	0.164

HsCRP	0.431	(0.586–1.223)	0.061

## Discussion

Our study has shown a high prevalence of SCH (21.9%) and overt hypothyroidism (7.4%) in patients with metabolic syndrome. In our control group 6.6% had SCH and 2% had overt hypothyroidism. A large multi-centered study on pediatric population from 10 states in India has shown an overall goiter prevalence of 4.78% with extremes of prevalence namely 31.02% and 0.02% being noted in 2 districts [16]. A recent review has identified the prevalence of sub-clinical hypothyroidism to be 4% to 8% in the general population, and up to 15% to 18% in women who were over 60 years of age in the western population [17]. However data is lacking with respect to the population prevalence of hypothyroidism in the adult South Indian population.

In our study mean systolic pressure, diastolic pressure, waist circumference, fasting blood sugar, total cholesterol, LDL cholesterol, triglycerides and TSH values were significantly higher in the MetS group compared to the control group. Our study has also shown a strong association between SCH and overt hypothyroidism with MetS. Similar to our observation the study by Uzunlulu et al. [18], had shown SCH prevalence to be 16.4% (n = 36) in the MetS group (n = 220). The MetS group in their study had significantly higher levels of mean systolic pressure, diastolic pressure, waist circumference, body mass index, fasting blood sugar, total cholesterol, LDL cholesterol, triglycerides and TSH values. SCH was significantly associated with MetS group (P = 0.001). However this study did not address patients with overt hypothyroidism and all observations were on SCH patients only.

Supporting our observations thyroid function has been consistently associated with individual components of metabolic syndrome. Recent studies have established the association between FT4 levels and total cholesterol, LDL cholesterol, HDL cholesterol, and triglycerides [19,20], and SCH is also associated with an increased level of Lipoprotein (a) [21]. The HUNT study concluded that "Within the range of TSH that is considered clinically normal, increasing level of TSH was associated with less favorable lipid concentrations. The association with serum lipids was linear across the entire reference range of TSH" [22].

With regard to other components of MetS, a low normal FT4 level was significantly associated with increased insulin resistance [19] and SCH has been associated with fasting hyperinsulinemia [23]. Diastolic arterial pressure has been significantly associated with TSH levels and T4 resistance-index (freeT4.TSH product) [24]. Hence in summary hypothyroidism is significantly associated with every individual component of metabolic syndrome.

In our study females with metabolic syndrome had significant association with SCH and overt hypothyroidism. The study by Uzunlulu et al. had also shown females to be associated with SCH and MetS [18]. A study from the United States has shown that among older white women, high TSH was associated with deleterious changes in serum lipids, particularly HDL-C, LDL-C, and the ratio of LDL-C to HDL-C cholesterol. Women with multiple lipid abnormalities were twice as likely to have an increased TSH [25]. The HYOGA study has shown that hypercholesterolemic women >50 years of age with SCH had symptoms of hypothyroidism and a poorer quality of life even when TSH was less than 10 mIU/L [26]. Hence it may be a good practice to screen females with metabolic syndrome for hypothyroidism.

In our study HsCRP was found to be associated with sub-clinical hypothyroidism in patients with metabolic syndrome. Previous published studies reflect a conflicting observation on the association between HsCRP and hypothyroidism. Tuzcu et al. and Christ-Crain et al. have shown a clear association between hypothyroidism and a raised HsCRP [23,27]. In contrary, Pearce et al. had shown that patients with Hashimoto's thyroiditis, short term hypothyroidism and post partum thyroiditis had similar HsCRP as compared to their euthyroid controls and that HsCRP levels may have only a limited role in the diagnosis of thyroid diseases [28]. The Study by Hueston et al. also showed no difference in HsCRP levels between patients with SCH and euthyroid individuals [29]. The important difference between these studies and our study is that all our study patients had metabolic syndrome whereas the other studies did not address patients with metabolic syndrome.

With respect to the association between vascular disease and raised HsCRP in hypothyroid patients, Nagasaki et al. showed that hypothyroid patients with a raised HsCRP have increased stiffness of the common carotid artery [30]. Similarly studies have shown HsCRP to be an additional risk factor for cardiovascular disease in hypothyroid patients [27]. The compounded cardiovascular risk that patients with metabolic syndrome, hypothyroidism and systemic inflammation (raised HsCRP) will suffer is yet to be determined. However Framingham off spring study had shown that the combined cardiovascular risk in patients with metabolic syndrome and a raised HsCRP were similar and not worse when compared to their individual risks [11].

The Tromso Study [20] and the Basel Thyroid Study [31] have shown that L-thyroxine replacement in patients with sub-clinical hypothyroidism has a beneficial effect on low density lipoprotein cholesterol levels and clinical symptoms of hypothyroidism. Also an important risk reduction in cardiovascular mortality of 9–31% can be estimated from the observed improvement in low density lipoprotein cholesterol [22,23]. Raising significant doubts about this estimated cardiovascular risk reduction a recent review which analyzed data from twelve trials conducted on levothyroxine replacement in sub-clinical hypothyroidism concluded that though thyroxine replacement improves some parameters in lipid profile and left ventricular function, this did not translate into improved survival and decreased cardiovascular morbidity [17]. However these trials did not address the issue of thyroxine replacement in patients with sub-clinical hypothyroidism in the setting of metabolic syndrome. Hence further randomized trials have to be planned in this line to determine the efficacy of thyroxine replacement in this group of patients.

Data is again conflicting with respect to the effect of thyroxine replacement on HsCRP levels in hypothyroid patients. In the study by Christ-Crain et al. thyroxine replacement did not alter HsCRP levels [27], whereas Nagasaki et al. observed a reduction of HsCRP levels with thyroxine replacement and it predicted improvement of arterial thickness in their study cohort [30].

## Conclusion

Sub-clinical and overt hypothyroidism is significantly associated with metabolic syndrome patients. Females have an increased risk of this association. Hence it will be worthwhile to screen female metabolic syndrome patients for hypothyroidism. MetS patients with SCH may have systemic inflammation and conversely, MetS patients with raised HsCRP are at risk for SCH. Whether this triple association between MetS, hypothyroidism, systemic inflammation will translate into compounded cardiovascular risk is yet to be determined. Controversy prevails with respect to benefits of thyroxine replacement in sub-clinical hypothyroidism. Whether metabolic syndrome patients with sub-clinical hypothyroidism will benefit from thyroxine replacement is for future randomized trials to answer.

## Abbreviations

NCEP – ATP III: National Cholesterol Education Program Adult Treatment Panel III; MetS: metabolic syndrome; SCH: sub-clinical hypothyroidism; LDL-C: low density lipoprotein cholesterol; HDL-C: high density lipoprotein cholesterol; HsCRP: high sensitivity C reactive protein; TSH: thyroid stimulating hormone; FT4: free T4; WHO: World Health Organization.

## Competing interests

The authors declare that they have no competing interests.

## Authors' contributions

GPSS, AAK, VJ, DR, SS were involved in concept, design of the study, acquisition of data, analysis and interpretation of data, review of literature, drafting and revising the manuscript. RK, KKS, SKN revised the manuscript for important intellectual content. All authors read and approved the final manuscript.

## References

[B1] Grundy SM (2006). Metabolic syndrome: connecting and reconciling cardiovascular and diabetes worlds. J Am Coll Cardiol.

[B2] Kylin E (1923). Studien ueber das Hypertonie-Hyperglyka "mie-Hyperurika" miesyndrom. Zentralblatt fuer Innere Medizin.

[B3] Reaven GM (1988). Banting lecture 1988. Role of insulin resistance in human disease. Diabetes.

[B4] World Health Organization (1999). Definition, diagnosis and classification of diabetes mellitus and its complications: report of a WHO Consultation. Part 1: diagnosis and classification of diabetes mellitus.

[B5] Tkac I (2005). Metabolic syndrome in relationship to type 2 diabetes and atherosclerosis. Diabetes Res Clin Pract.

[B6] Mohan V, Deepa M, Farooq S, Datta M, Deepa R (2007). Prevalence, Awareness and Control of Hypertension in Chennai – The Chennai Urban Rural Epidemiology Study (CURES – 52). JAPI.

[B7] Gupta R, Deedwania PC, Gupta A, Rastogi S, Panwar RB, Kothari K (2004). Prevalence of metabolic syndrome in an Indian urban population. Int J Cardiol.

[B8] (2002). American Association of Clinical Endocrinologists medical guidelines for clinical practice for the evaluation and treatment of hyperthyroidism and hypothyroidism. Endocr Pract.

[B9] Dillmann WH (1985). Mechanism of action of thyroid hormones. Med Clin North Am.

[B10] Serter R, Demirbas B, Culha C, Cakal E (2004). The effect of L-thyroxine replacement therapy on lipid based cardiovascular risk in sub clinical hypothyroidism. Invest J Endocrinol.

[B11] Rutter MK, Meigs JB, Sullivan LM, D'Agostino RB, Wilson PW (2004). C-reactive protein, the metabolic syndrome, and prediction of cardiovascular events in the Framingham Offspring Study. Circulation.

[B12] Taki K, Nishio K, Hamajima N, Niwa T (2008). Metabolic syndrome defined by new criteria in Japanese is associated with increased liver enzymes and C-reactive protein. Nagoya J Med Sci.

[B13] Executive summary of the Third Report of the National Cholesterol Education Program (NCEP) Expert Panel (2001) on Detection, Evaluation and Treatment of high Blood Cholesterol in Adults (Adult Treatment Panel III). JAMA.

[B14] Friedewald WT, Levy RI, Friedrickson DS (1972). Estimation of the concentration of lowdensity lipoprotein in plasma without the use of preparative ultracentrifuge. Clin Chem.

[B15] Ross DS (2001). Serum thyroid – stimulating hormone measurement for assessment of thyroid function and disease. Endocrinol Metab Clin North Am.

[B16] Toteja GS, Singh P, Dhillon BS, Saxena BN (2004). Iodine deficiency disorders in 15 districts of India. Indian J Pediatr.

[B17] Villar HC, Saconato H, Valente O, Atallah AN (2007). Thyroid hormone replacement for subclinical hypothyroidism. Cochrane Database Syst Rev.

[B18] Uzunlulu M, Yorulmaz E, Oguz A (2007). Prevalance of subclinical hypothyroidism in patients with metabolic syndrome. Endocr J.

[B19] Roos Annemieke, Bakker StephanJL, Links TheraP, Gans RijkOB, Wolffenbuttel BruceHR (2007). Thyroid Function Is Associated with Components of the Metabolic Syndrome in Euthyroid Subjects. J Clin Endocrinol Metab.

[B20] Iqbal A, Jorde R, Figenschau Y (2006). Serum lipid levels in relation to serum thyroid-stimulating hormone and the effect of thyroxine treatment on serum lipid levels in subjects with subclinical hypothyroidism: the Tromsø Study. J Intern Med.

[B21] Kung AW, Pang RW, Janus ED (1995). Elevated serum Lipoprotein (a) in sub-clinical hypothyroidism. Clin Endocrinol (Oxf).

[B22] Asvold BO, Vatten LJ, Nilsen TI, Bjøro T (2007). The association between TSH within the reference range and serum lipid concentrations in a population-based study. The HUNT Study. Eur J Endocrinol.

[B23] Tuzcu A, Bahceci M, Gokalp D, Tuzun Y, Gunes K (2005). Subclinical hypothyroidism may be associated with elevated high-sensitive c-reactive protein (low grade inflammation) and fasting hyperinsulinemia. Endocr J.

[B24] Saltiki K, Voidonikola P, Stamatelopoulos K, Mantzou E, Papamichael C, Alevizaki M (2008). Association of thyroid function with arterial pressure in normotensive and hypertensive euthyroid individuals: A cross-sectional study. Thyroid Res.

[B25] Bauer DC, Ettinger B, Browner WS (1998). Thyroid functions and serum lipids in older women: a population-based study. Am J Med.

[B26] Leclère J, Cousty C, Schlienger JL, Wémeau JL (2008). Subclinical hypothyroidism and quality of life of women aged 50 or more with hypercholesterolemia: results of the HYOGA study. Presse Med.

[B27] Christ-Crain M, Meier C, Guglielmetti M, Huber PR, Riesen W, Staub JJ, Müller B (2003). Elevated C-reactive protein and homocysteine values: cardiovascular risk factors in hypothyroidism? A cross-sectional and a double-blind, placebo-controlled trial. Atherosclerosis.

[B28] Pearce EN, Bogazzi F, Martino E, Brogioni S, Pardini E, Pellegrini G, Parkes AB, Lazarus JH, Pinchera A, Braverman LE (2003). The prevalence of elevated serum C-reactive protein levels in inflammatory and noninflammatory thyroid disease. Thyroid.

[B29] Hueston WJ, King DE, Geesey ME (2005). Serum biomarkers for cardiovascular inflammation in subclinical hypothyroidism. Clin Endocrinol (Oxf).

[B30] Nagasaki T, Inaba M, Shirakawa K, Hiura Y, Tahara H, Kumeda Y, Ishikawa T, Ishimura E, Nishizawa Y (2007). Increased levels of C-reactive protein in hypothyroid patients and its correlation with arterial stiffness in the common carotid artery. Biomed Pharmacother.

[B31] Meier C, Staub JJ, Roth CB, Guglielmetti M, Kunz M, Miserez AR, Drewe J, Huber P, Herzog R, Müller B (2001). TSH-controlled L-thyroxine therapy reduces cholesterol levels and clinical symptoms in subclinical hypothyroidism: a double blind, placebo-controlled trial (Basel Thyroid Study). J Clin Endocrinol Metab.

